# Metabolic complications during HIV-treatment with protease inhibitors in patients from Braşov

**DOI:** 10.1186/1471-2334-14-S4-P32

**Published:** 2014-05-29

**Authors:** Maria-Elena Cocuz, Rodica Silaghi, Bianca Nedelcu, Geta Manea

**Affiliations:** 1 Faculty of Medicine, Transilvania University, Braşov, Romania; 2 Infectious Diseases Hospital, Braşov, Romania

## 

Objective: to evaluate the effects of treatment with protease inhibitors on serum level of triglycerides, cholesterol and glycemia in HIV-infected patients, in assessing the need of monitoring patients.

We performed a retrospective study on 25 patients with HIV infection, in evidence of the Infectious Diseases Hospital of Braşov, treated with protease inhibitors in the treatment regimen. We studied the levels of cholesterol, serum triglycerides and blood glucose at the beginning and during treatment with protease inhibitors.

We found increases in serum cholesterol levels in 72% of patients and in 83.33% of them were increasing by 50% from baseline; growth exceeded baseline in 44.45% of cases. Serum triglycerides have increased to 68% of patients, with up to 100% from baseline in 64.5% of cases and in the rest of the patients the increase was marked, beyond 100% exceeded normal values in 64.5% of cases. Blood glucose was maintained at normal levels in 56% patients and increased in 24% of cases.

Treatment with protease inhibitors is associated frequently with increased serum levels of cholesterol and triglycerides; hyperglycemia has rarely occurred. Patients treated with protease inhibitors require rigorous monitoring of the metabolism of fats and carbohydrates in order to establish appropriate treatment for early complications that may have occurred.

